# Nutritional recommendations for patients undergoing prolonged glucocorticoid therapy

**DOI:** 10.1093/rap/rkac029

**Published:** 2022-04-21

**Authors:** Gabriel P Esteves, Bruna Caruso Mazzolani, Fabiana Infante Smaira, Elizabeth Silva Mendes, Gabriela Guimarães de Oliveira, Hamilton Roschel, Bruno Gualano, Rosa Maria R Pereira, Eimear Dolan

**Affiliations:** 1 Applied Physiology & Nutrition Research Group; School of Physical Education and Sport; Rheumatology Division; Faculdade de Medicina FMUSP; 2 Bone Metabolism Laboratory, Rheumatology Division; Faculdade de Medicina FMUSP, Universidade de Sao Paulo, Sao Paulo, Brazil

**Keywords:** prednisone, corticosteroid, diet, nutrition, food, lifestyle

## Abstract

Glucocorticoid (GC) therapy is a common treatment used in rheumatic and autoimmune diseases, owing to its anti-inflammatory and immunosuppressive effects. However, GC therapy can also induce a number of adverse effects, including muscle and bone loss, hypertension, metabolic perturbations and increased visceral adiposity. We review available evidence in this area and provide nutritional recommendations that might ameliorate these adverse effects. Briefly, optimizing calcium, vitamin D, sodium and protein intake and increasing consumption of unprocessed and minimally processed foods, while decreasing the consumption of ultra-processed foods, might counteract some of the specific challenges faced by these patients. Importantly, we identify a dearth of empirical data on how nutritional intervention might impact health-related outcomes in this population. Further research is required to investigate the clinical and therapeutic efficacy of these theory-based recommendations.

Key messages

- Glucocorticoid therapy can cause bone and muscle loss, metabolic dysregulation, visceral fat accumulation and hypertension.
- Optimizing calcium, vitamin D, protein, sodium and food processing level may attenuate these adverse effects.
- High-quality studies are necessary to confirm the efficacy of these theory-based recommendations.

## Introduction

**Fig. 1:**
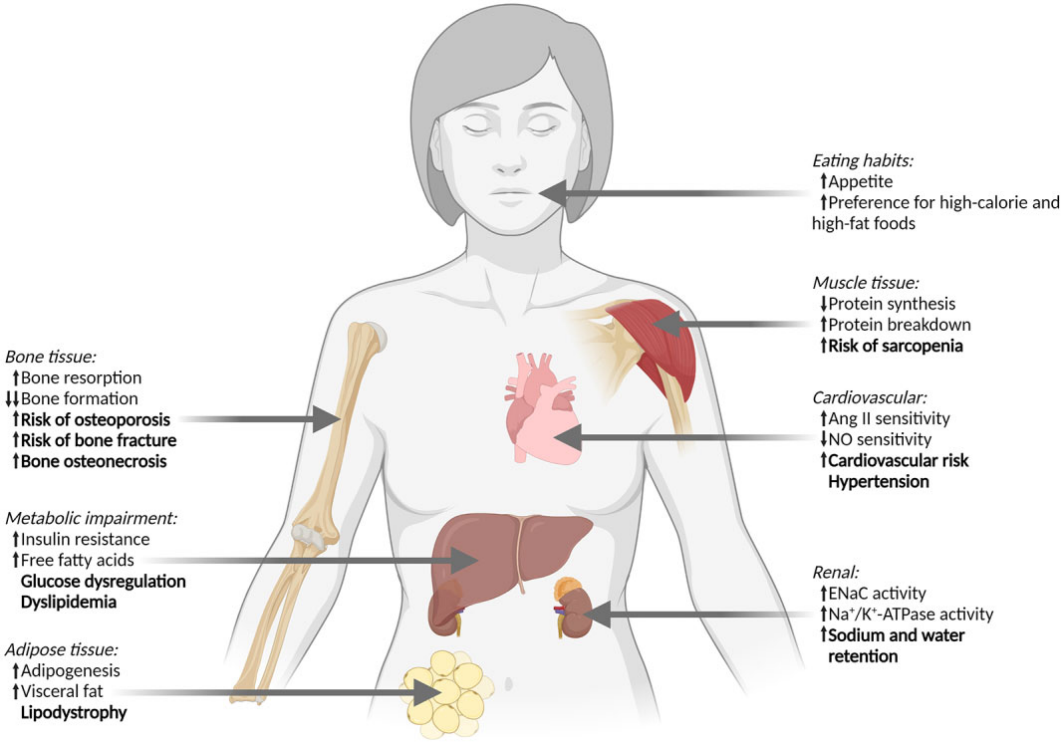
Adverse effects attributable to prolonged glucocorticoid therapy Prolonged glucocorticoid therapy has important adverse effects in many tissues. These effects include: bone and muscle loss, which increases the risk of bone fractures and sarcopenia; metabolic impairments, which can lead to glucose and lipid dysregulation; increases in adipose tissue and visceral fat, alongside abnormal fat distribution; increased appetite and preference for high-calorie foods; and increased water and sodium retention, alongside increased blood pressure and risk for cardiovascular diseases. Mechanisms are presented in normal text, clinical outcomes in bold text. Ang II: angiotensin II; ENaC: epithelial Na^+^ channel; NO: nitric oxide.

Glucocorticoids (GCs) are one of the most widely prescribed family of medications available and have myriad clinical applications [1], which relate primarily to their ability to up-regulate anti-inflammatory and down-regulate pro-inflammatory pathways [2]. This treatment strategy has been reported to be effective in many autoimmune diseases, such as SLE and RA, and in other conditions, such as adrenal insufficiency, cancer, allergies, asthma and skin diseases. Despite these clinical applications, prolonged GC therapy has a number of adverse effects (Fig. 1) [3, 4]. These effects include bone and muscle loss and dysfunction; metabolic perturbations, such as dyslipidaemia and glucose dysregulation; and excessive and abnormal fat accrual [1, 5]. These adverse effects are dose related [6] and are particularly concerning for individuals whose chronic conditions necessitate prolonged treatment.

Specific nutritional strategies have the potential to prevent or attenuate many of these GC-induced adverse effects, but there is currently a paucity of information as to what these nutritional recommendations should comprise, rendering it difficult for health-care professionals to provide targeted and evidence-based advice to their patients. The aim of the present paper, therefore, is to provide a narrative review of available evidence related to the adverse effects associated with GC therapy and to provide practical nutritional recommendations that might counteract or ameliorate these issues.

## The influence of GC therapy on bone and skeletal muscle

### Bone tissue

The adverse effects of prolonged GC use on bone are well documented, with the most common cause of secondary osteoporosis being GC therapy [7, 8]. The influence of GCs on bone seems to manifest initially as an increase in bone resorption within the first 5–7 months of treatment [9], whereas in the longer term, reduced bone formation is believed to be the primary driver of GC-induced bone loss [10]. Meta-analytical data indicate that GC use (≥ 5 mg·day^−1^ prednisone or equivalent) for >3 months can lead to a 2- and 3-fold increase of hip and vertebral fracture risk, respectively [11]. As such, strategies to protect bone are an important goal of many GC therapy regimens, and nutritional intervention has substantial potential to help achieve this goal.

A range of nutrients are required to maintain bone health [12, 13], with calcium and vitamin D being particularly relevant. Calcium accounts for ∼1–2% of human body mass, with 99% of this found in mineralized tissues, such as bone. This nutrient plays a pivotal role in both bone structure and BMD growth during early development, and in preventing its decay during later years [14]. Vitamin D, a fat-soluble secosteroid, is another important component implicated in bone health, mainly owing to its capacity to increase calcium absorption in the small intestine [15]. The importance of vitamin D to adequate bone structure and growth is exemplified by conditions characterized by its deficiency, such as rickets and osteomalacia, whereby defective mineralization culminates in deformed structure (bowing) and increased fracture risk [16]. As such, adequate intake of calcium and vitamin D is essential to any nutritional intervention aiming to support and improve bone health (for an overview of all nutritional recommendations, see Table 1). This is particularly relevant for patients undergoing GC therapy, because this treatment strategy can also lead to decreased intestinal absorption of calcium, along with increased renal excretion [7, 17], both of which can perturb calcium homeostasis further.

**Table 1:** Summary of nutritional recommendations to reduce adverse effects attributable to prolonged glucocorticoid therapy

Organ/system	Adverse effects	Nutritional recommendations
Bone tissue	↑ bone resorption and ↓↓ bone formation [10] ↓ intestinal calcium absorption [7, 17] ↑ urinary calcium excretion [7, 17] ↑ risk of osteoporosis and bone fractures [7, 11]	Optimize calcium intake to 1000–1200 mg·day^−1^ [17] Optimize vitamin D intake to 600–800 UI·day^−1^ [17, 20] Maintain optimal protein intake [44, 45]
Muscle tissue	↓ protein synthesis [56, 57] ↑ skeletal muscle autophagy [56, 57] ↓ muscle mass and force [58] ↑ risk of sarcopenia [60]	No chronic kidney disease: optimize intake of high-quality proteins to 1.0–1.5 g·kg^−1^·day^−1^ [62, 63] Chronic kidney disease stages 3–5: maintain high-quality protein intake at 0.6 g·kg^−1^·day^−1^ [66]
Body weight, lipid profile and glucose homeostasis	↑ adipogenesis [72, 77] ↑ visceral fat [78] ↑ weight gain [77] ↑ insulin resistance [79] Lipodystrophy [75] Dyslipidaemia [73]	↑ unprocessed/minimally processed food intake [101, 103–106] ↓ ultra-processed food intake [101, 103–107] Energy balance: base daily requirements on estimated resting metabolic rate and physical activity level Energy restriction: aim for moderate energy restrictions when needed [117]
Renal/cardiovascular	↑ sodium and water retention [73] ↑ blood pressure [73] ↑ risk of hypertension and cardiovascular disease [122, 123]	Maintain adequate sodium intake (<1500 mg·day^−1^) [125] ↑ unprocessed/minimally processed food intake [130] ↓ ultra-processed food intake [130]

Dietary reference values indicate the recommended daily nutrient intake for the general population. Calcium intakes of ∼950–1000 mg·day^−1^ are recommended for healthy adults [18, 19], while US guidelines indicate that this should be increased to 1200 mg·day^−1^ for adults aged >70 years [19, 20]. Vitamin D is obtained primarily through endogenous synthesis after exposure to sunlight [21], but can also be obtained through dietary ingestion. Current guidelines recommend an oral intake of vitamin D of 600 UI·day^−1^ (15 µg) for healthy adults, increasing to 800–1000 UI·day^−1^ (20 µg) in adults aged >70 years [19, 22]. These recommendations were developed assuming minimal sunlight exposure, thus ensuring a value that is inclusive of most populations [19, 22]. This is particularly relevant for patients undergoing prolonged GC therapy (e.g. patients with SLE or skin cancer), because they may be required to restrict sunlight exposure owing to photosensitivity and potential induction of disease activity [23]. As such, adequate vitamin D intake, be it via whole foods or supplements, can be particularly important to ensure adequate status in this population.

Current guidelines from the ACR for prevention and treatment of GC-induced osteoporosis align with these dietary guidelines and recommend that patients aim for the upper level of recommended intakes of calcium (1000–1200 mg·day^−1^) and vitamin D (600–800 UI·day^−1^) [17, 20]. It is important to highlight, however, that these are classified as ‘conditional recommendations’ [17], meaning that the available evidence points towards benefits of this intervention most likely outweighing its potential undesirable effects, although evidence is still limited. Considering data from the general population, meta-analytical estimates based on young adults and institutionalized individuals indicate that calcium and vitamin D supplementation can reduce the risk of total fractures [relative risk (RR) = 0.86] and hip fractures (RR = 0.61) [24, 25], although the micronutrient status at baseline is likely to be an important determinant of response to supplementation. For example, a recent sub-cohort study from a larger clinical trial showed that the supplementation of vitamin D did not improve BMD in healthy individuals with adequate vitamin D status, but a small increase in spine BMD was observed within individuals who had a lower free 25(OH)vitamin D status at baseline [26]. This is particularly relevant for individuals undergoing GC treatment, given the aforementioned issues related to calcium absorption and sunlight exposure, which may increase the risk of calcium and vitamin D deficiency, accompanied by the high risk for GC-induced bone loss. Evidence related to the efficacy of calcium and vitamin D supplementation on bone parameters in this population is, however, somewhat mixed. It seems that calcium supplementation alone does not prevent GC-induced BMD declines in patients with rheumatic and immunological diseases [27]. The co-supplementation of calcium and vitamin D, however, has been shown to be effective at preserving BMD at the lumbar spine and trochanter during GC therapy in a randomized clinical trial of RA patients [28] and at the lumbar spine in a meta-analysis of nine trials, which included patients who had a range of rheumatic conditions [29]. Regarding the prevention of fracture rates, current data on this topic currently come from sub-analyses of two meta-analyses, both of which included two studies only [30, 31], and from the summary of findings of the 2017 ACR guidelines for GC-induced osteoporosis management [17], which included three outcomes from two studies. In most studies, the point estimate did favour vitamin D supplementation (with or without calcium) over placebo, but the uncertainty was too large to conclusively determine the efficacy of this dietary approach. As such, further studies are needed to provide an accurate answer to the question of whether co-supplementation of calcium and vitamin D can prevent fractures in patients undergoing GC therapy.

Notwithstanding the need for further investigation of the efficacy of calcium and/or vitamin D supplementation on bone health and fracture risk in individuals who undergo GC treatment, the importance of these micronutrients for bone health is clear, and maintaining adequate status should be a priority within nutritional plans (see Tables 1 and 2). Where possible, we recommend that all micronutrient requirements should be met primarily using a food-first approach and that nutrients should be obtained through whole foods rather than supplements [8, 32] (Table 2). The benefits of this approach are many. For instance, the risk of ingesting toxic levels of micronutrients through the diet is significantly lower than with oral supplements. Also, the consumption of whole foods, rather than isolated nutrients, will be likely to improve the nutritional status of a wide range of nutrients, and not only the micronutrient of interest [33]. Calcium is widely available in dairy products (e.g. milk, cheese and yogurt) and dark leafy vegetables [34], whereas vitamin D is found primarily in oily fishes, such as trout, tuna and salmon [34]. Although it is relatively easy to achieve adequate calcium intake via diet alone, the same might not hold true for vitamin D, particularly in countries where fish consumption is less common. In such cases, and when sunlight exposure is inadvisable or insufficient, vitamin D supplementation might be important to achieve these recommendations.

**Table 2:** Practical examples of food portions necessary to meet nutritional recommendations

Nutritional recommendation	Nutrient food source	Portions to meet recommendation
Calcium: 1000–1200 mg·day^−1^	Milk, skimmed	2 cups (380 g)
	Yogurt, plain, low fat	8 ounces (225 g)
	Cheese, mozzarella	2 ounces (55 g)
	Spinach, cooked	½ cup (90 g)
Vitamin D: 600–1000 UI·day^−1^	Milk, skimmed	2 cups (380 g)
	Yogurt, plain, low fat	8 ounces (225 g)
	Salmon, grilled	3 ounces (85 g)
	Sardines, canned	3 ounces (85 g)
Protein: 1–1.5 g·kg^−1^·day^−1^	Milk, skimmed	2 cups (380 g)
	Yogurt, plain, low fat	8 ounces (225 g)
	Salmon, grilled	3 ounces (85 g)
	Chicken leg, roasted	4 ounces (110 g)
	Lentils, cooked	2 ounces (55 g)
Protein (chronic kidney disease stages 3–5): 0.6–0.8 g·kg^−1^·day^−1^*	Milk, skimmed	2 cups (380 g)
	Yogurt, plain, low fat	8 ounces (225 g)
	Chicken leg, roasted	2 ounces (56 g)
	Lentils, ripe seed, cooked, with salt	1 ounce (28 g)
Sodium: <1500 mg·day^−1^	Salt distributed throughout meals	3.75 g (½ teaspoon)

This represents a practical sample menu only, and prescriptions should be adapted for each individual. Nutrient intakes using these portions are likely to be higher than presented, because only main sources were accounted for. Source: USDA FoodData Central, US Department of Agriculture.

* It is important to note that these recommendations constitute a protein restriction and should be implemented when medically advised and under the care of a certified dietitian, nutritionist or international equivalent.

Although calcium and vitamin D are widely recognized as essential nutrients for bone health, it is important to highlight that a myriad of other nutrients are also implicated in bone metabolism and calcium homeostasis [33]. Micronutrients such as phosphorus [35] and vitamin C [36] are part of the bone formation and mineralization processes, while potassium [37] and magnesium [38] are involved in calcium homeostasis. Vitamin K also seems to exert potentially protective effects on bone [39]. Adequate nutritional status of these micronutrients [40–42] has been associated with improved BMD in adult men and women [36–39, 43]. As described previously, these diverse micronutrients are available across different food types, and as such, adequate intake is best achieved through a diet consisting of natural, nutrient-dense foods, such as fruits, vegetables, nuts, dairy and lean protein sources [33]. Adequate protein intake might also be beneficial for bone health [44–47]. Meta-analytical findings indicate a significant, albeit small, positive effect of higher protein intake on BMD [48–50], along with reduced risk of fractures [50, 51]. Importantly, a meta-analysis investigating protein intakes that exceeding the minimal requirement of protein intake (i.e. >0.8 g·kg^−1^·day^−1^) showed a significant decrease in hip fractures in healthy adults [52]. Considerations and recommendations for protein intake in individuals undergoing GC therapy are described in the next section regarding skeletal muscle.

Finally, adequate energy intake is essential to protect bone health. When available energy is insufficient to support all biological processes simultaneously, down-regulation of certain processes can occur [53], including bone formation [54, 55]. This might be particularly relevant for individuals undergoing prolonged GC use, given that weight gain and visceral fat accumulation are common in these patients, and some dietary restriction is often recommended to avoid this. Certainly, this might be prudent in some situations, but it is important that dietary restriction is not taken too far, lest other processes, such as bone metabolism, be impacted negatively. This topic will be discussed in more detail in the section regarding the influence of GC therapy on body composition, lipid and glucose metabolism, along with recommendations to maintain adequate energy availability and, in turn, avoid the potentially negative implications associated with energy imbalance (be it a surplus or a deficit).

### Skeletal muscle

Skeletal muscle mass is ultimately maintained, lost or increased based upon a dynamic balance between muscle protein synthesis and breakdown. Current evidence indicates that GCs lead to an increase in muscle protein breakdown by activating the ubiquitin–proteasome and lysosomal systems, while also decreasing protein synthesis by disrupting adequate cell signalling of important growth factors, such as insulin and insulin like growth factor 1 (IGF1; Fig. 1) [56, 57]. These mechanistic data are reinforced by studies on human subjects, which have shown that dexamethasone (a commonly prescribed GC) administered for 1 week led to decreased muscle fibre cross-sectional area and excitability, induced myosin loss and reduced force in healthy participants [58, 59]. Additionally, in a cohort of RA patients followed for 1 year, treatment with GCs was associated with sarcopenia (*r* = 0.25), and doses higher than 3.25 mg·day^−1^ were identified as an important independent risk factor for sarcopenia (odds ratio = 8.11) [60].

Appropriate protein intake is essential to support muscle anabolism, and current guidelines for healthy adults recommend a protein intake of ∼0.8 g·kg^−1^·day^−1^ [52, 61], which would equate to an intake of ∼56 and ∼46 g·kg^−1^·day^−1^ for an individual who weighs 70 or 57.5 kg. Intakes greater than these are, however, advised for populations who are susceptible to muscle loss, such as older adults [62] or cancer patients [63]. Given that GCs convey a catabolic stimulus that increases risk of muscle loss [64], patients undergoing this treatment strategy might also benefit from protein intakes above the current guidelines. To the best of our knowledge, no study has directly investigated the therapeutic potential of increasing protein intake in this population. Pending such evidence, we recommend aligning with guidelines provided to other populations at risk of muscle loss or sarcopenia [62, 63], which is to aim for protein intakes of ∼1.0–1.5 g·kg^−1^·day^−1^ (Table 2). It is important to acknowledge that this recommendation is based on studies investigating other clinical populations and that specific studies aimed at understanding the role of protein intake in individuals undergoing GC therapy are warranted (Table 3).

**Table 3:** Potential key research questions related to diet and glucocorticoid therapy

Organ/topic	Key research questions
Bone tissue	Can vitamin D and calcium supplementation reduce fracture risk in patients undergoing GC therapy?
	Are higher protein intakes (>0.8 g·kg^−1^·day^−1^) beneficial for the bone health of patients undergoing GC therapy?
Muscle tissue	What are the clinical effects of glucocorticoid treatment on muscle mass and function and does this relate to dosage?
	Are higher protein intakes (>0.8 g·kg^−1^·day^−1^) beneficial for muscle mass and function in patients undergoing GC therapy?
Lipid and glucose homeostasis	What are feasible and effective dietary patterns and holistic interventions to improve lipid profile and glucose homeostasis on patients undergoing GC therapy?
Renal/cardiovascular	Can adequate sodium intake aid in reducing the prevalence of hypertension in patients undergoing GC use?
	What dietary patterns are feasible and effective ways to improve blood pressure in this population?
GC therapy effects on eating behaviour	How does glucocorticoid therapy influence dietary patterns and eating behaviour?
	What are the motivations associated with increases or decreases in food consumption and food choice?

GC: glucocorticoid.

In addition to consuming adequate quantities of protein to support muscle anabolism, the type of protein must also be considered [63]. Protein quality is classified according to two important factors, namely the presence of essential amino acids, which are those not synthesized by the body and that must be ingested through the diet, and protein digestibility (i.e. how efficiently proteins are digested and amino acids absorbed by the small intestine) [64]. High-quality proteins, therefore, contain adequate and bioavailable amounts of all essential amino acids and are obtained primarily through animal source foods, such as meat, fish, eggs and dairy, but also through plant source foods, such as soybean, isolated plant-based proteins (e.g. soy protein concentrate and pea protein concentrate), or through the combination of different plant source foods [63, 65, 66].

Although protein intakes of the magnitude recommended herein (i.e. 1.0–1.5 g·kg^−1^·day^−1^) are considered safe in healthy adults [65], some caution must be applied for individuals with conditions that impact renal health, given that adequate kidney function is required to process and eliminate the waste products of protein metabolism [66]. This might be particularly relevant for patients undergoing GC therapy, given that some might simultaneously present with renal complications. For example, patients with SLE might present with lupus nephritis, a renal manifestation of the disease [67]. More broadly, GCs are a frequent treatment for patients with glomerulonephritis owing to their immunosuppressive effects [68]. In such conditions, an individualized approach that considers the stage and severity of kidney disease is necessary, and the goal of nutrition therapy should be shifted, focusing on preservation of kidney function [69]. Therefore, in line with recent guidelines, we recommend that patients undergoing GC therapy who also present with chronic kidney disease stages 3–5 (defined as glomerular filtration rate <60 ml·min^−1^ and albuminaemia >3 mg·mmol^−1^ [70]) maintain protein intake at 0.6 g·kg^−1^·day^−1^ [66]. The supplementation with amino acid keto-analogues (nitrogen-free analogues of the main essential amino acids) might also have a role in the nutritional management of kidney disease [71], allowing for very low whole protein intakes (i.e. 0.28–0.43 g·kg^−1^·day^−1^), while also possibly preventing malnutrition [66]. It is important to note that both these recommendations constitute a protein restriction and are indicated only for CKD patients at an advanced disease stage and who are not undergoing dialysis treatment. As such, they should be implemented only when medically advised by a certified dietitian nutritionist or international equivalent. Further information and guidance can be found in the original Kidney Disease Outcomes Quality Initiative (KDOQI) publication [66].

## The influence of GC therapy on body composition, lipid and glucose metabolism

GCs exert a number of systemic metabolic effects, and their prolonged use can eventually contribute to lipid [72, 73] and glucose [74] dysregulation, increased visceral fat and increased risk of central obesity and metabolic disorders [75, 76]. GCs act on the adipose tissue by increasing lipid synthesis and storage, promoting adipocyte hypertrophy and increasing adipogenesis by stimulating pre-adipocyte differentiation into mature adipocytes [72, 77]. These alterations are more pronounced in visceral adipose tissue, which has a higher density of GC receptors in comparison to subcutaneous adipose tissue [78]. GCs can also contribute to impaired glucose metabolism via a range of mechanisms, which are discussed in detail elsewhere [79]. One of the principal roles of endogenous GCs is to increase substrate availability during times of stress, such as glucose and free fatty acids [80]. This is achieved, for instance, by increasing liver gluconeogenesis and decreasing glucose uptake by the muscle, or by increasing lipolysis during acute increases in GC, such as during exercise [81]. Chronic activation of these pathways, however, can lead to glucose dysregulation, which manifests as increased insulin resistance and hyperglycaemia [79]. Collectively, these effects can increase the risk of type 2 diabetes or worsen glycaemic control in individuals already diagnosed with this condition [73].

In addition to these direct effects on lipid and glucose metabolism, GCs can contribute indirectly to unhealthy weight gain and metabolic perturbations by stimulating appetite and increasing preference for high-calorie, high-fat foods [73]. Between 60 and 70% of patients report weight gain after long-term use of GC [82], and two-thirds develop lipodystrophy [75], a modification of the fat accumulation pattern reminiscent of a Cushingoid pattern, which is associated with dyslipidaemia and cardiovascular disease [76, 83]. A systematic review that synthesized available evidence regarding the influence of GC use on energy intake, energy expenditure and body weight confirmed that short-term GC therapy leads to increased energy intake, but also to an increase in energy expenditure [77]. Clinically significant increases in body weight (i.e. >5% increase in body weight) were seen only in long-term therapy [77], suggesting that the weight gain associated with GC use depends on the duration of treatment. These alterations in weight and adipose tissue, when considered in the context of the previously discussed bone and muscle loss, might contribute to an osteosarcopenic obese phenotype, which might have important adverse health consequences [84].

Collectively, the metabolic effects of exogenous GC therapy can increase the risk of obesity [85, 86], diabetes [86–88], dyslipidaemia [86, 89] and associated cardiovascular diseases [90, 91]. All these conditions are, however, amenable to nutritional therapy, and as such, targeted nutritional recommendations might ameliorate many of these adverse consequences. Patients who are undergoing GC therapy, along with their health-care providers, should remain cognisant of the importance of consuming nutrient-dense and energetically balanced diets. The association of single nutrients with health-related parameters (e.g. carbohydrate intake and type 2 diabetes; fat intake and cardiovascular diseases) has been deemed overly reductionist [92, 93]; hence, more holistic approaches might be preferable. For example, dietary patterns such as plant-based diets or the Mediterranean diet are based primarily on natural, whole, minimally processed foods, and both are associated with improved cardiovascular health across the general population [94, 95]. Food processing level represents a holistic approach to nutritional intervention and is emerging as a promising means of categorizing the overall nutritional quality of the diet [93, 96]. NOVA is a classification system that categorizes foods based on processing level and is used as a tool to understand diet quality and to develop public health research and health policies [96]. It categorizes foods in four distinct types, namely: unprocessed or minimally processed foods; culinary ingredients; processed foods; and ultra-processed foods (see the supplementary table available in the paper by Monteiro *et al.* [97] for in-depth descriptions of NOVA food classification and examples).

Ultra-processed foods tend to be energy dense and highly palatable, typically leading to excess intake of fat, sugar and salt and lower intake of fibre, protein, vitamins and minerals [98–101]. In observational studies, a higher consumption of ultra-processed foods has been associated with increased cardiovascular risk and occurrence of chronic diseases such as diabetes, dyslipidaemia and obesity [102–105]. Conversely, higher consumption of unprocessed and minimally processed foods has been associated with lower risk of the same conditions [101, 106]. Furthermore, a randomized clinical trial showed that individuals consuming an ultra-processed food-rich diet significantly increased their energy intake and body weight within a 2-week period when compared with individuals on a diet of unprocessed or minimally processed foods [107]. As such, dietary guidelines from many countries, such as Brazil [108], Peru [109], Ecuador [110] and Israel [111], recommend basing the diet on unprocessed or minimally processed foods, while simultaneously minimizing ultra-processed food intake. Although yet to be tested directly with patients who are undergoing GC use, this dietary strategy seems to be a sensible option, given that it has the capacity to tackle many of the aforementioned adverse effects on lipid and glucose metabolism. Unprocessed foods such as meats, eggs, milk, legumes and vegetables also tend to be rich in nutrients essential to bone and muscle and, as such, might also help to alleviate the aforementioned negative musculoskeletal consequences of GC therapy.

Although GC-associated weight gain and visceral fat accumulation are problematic and might be alleviated with appropriate nutrition intervention, it is important to highlight that excessive dietary or energy restriction is ill advised during GC therapy. Adequate energy intake is essential to maintain function of all body systems and processes, and when energy availability is low, the body may selectively down-regulate certain processes (e.g. bone metabolism [53, 112, 113]) to conserve energy for processes deemed to be more immediately essential to survival. Individual energy requirements are likely to vary widely and to depend on factors including the patient’s age, sex, physical activity level and clinical status. An approximate indication of an individual’s energy needs can be estimated by considering their resting metabolic rate and physical activity level. For example, an individual who is sedentary or engages in light physical activity will expend ∼1.4–1.5 times their resting metabolic rate. As such, a 40-year-old woman with height of 165 cm and who weighs 70 kg will have a resting metabolic rate of ∼1443 kcal (calculated using the Harris–Benedict equation [114]). Assuming a physical activity level of 1.4–1.5, she should consume ∼2000–2160 kcal to meet her daily energy demands. In the event that an individual is consuming substantially more than their estimated requirements and when weight loss is required for health purposes, a reduction in energy intake may be advised. Moderate restrictions from their current energy intake (e.g. ∼500–1000 kcal·day^−1^), intended to bring about gradual and sustainable weight loss, are recommended. Given that diets rich in ultra-processed foods tend to lead to increased energy intake [107], moderate reductions in energy intake can often be achieved by reducing ultra-processed food intake and increasing unprocessed and minimally processed food intake, which is an achievable goal for many patients. Studies comparing rapid *vs* more gradual weight-loss interventions have shown similar efficacy in relationship to weight loss [115, 116], but more severe energy deficits might have other health-related consequences. For example, severe energy restriction (65–75% of estimated energy expenditure) led to a greater loss of hip bone mineral density compared with a more conservative energy restriction (25–35%) intervention [117] in a group of obese postmenopausal women. Given that patients undergoing GC therapy are already at high risk of bone and muscle loss, severe energy or nutrient restriction is ill advised. To reiterate the point made earlier, however, estimation of energy requirement is complex and multi-factorial. All numerical recommendations described herein are approximate estimates, and individual requirements can vary considerably owing to factors such as body composition, activity levels and clinical status [118].

## The influence of GC therapy on fluid and electrolyte balance

Sodium and water retention, leading to hypertension, are commonly cited adverse effects related to GC therapy [73]. This can be explained by the vascular effects of GCs, which include increased sensitivity to pressor agents, such as angiotensin II and catecholamines, and reduced sensitivity to vasodilators, such as nitric oxide [119]. GCs are also known to interact with the mineralocorticoid receptor, therefore mimicking the role of aldosterone [120] and increasing renal sodium and water retention (Fig. 1). Collectively, this combined influence of increased vasoconstriction, alongside increased fluid retention, can lead to an increase in blood pressure, which, if sustained in the long term, can have adverse cardiovascular consequences [121]. Indeed, a prospective study of patients with RA reported that long-term exposure to a high GC dosage (≥7.5 mg·day^−1^ prednisolone) was associated with higher prevalence of hypertension [122], and further meta-analytical data indicate a 2.19 odds ratio for development of hypertension in patients undergoing GC therapy compared with placebo [123].

Sodium is a micronutrient closely related to cardiovascular health and hypertension management. Although sodium intake recommendations for healthy adults range from 1500 to 2400 mg·day^−1^ [40, 124], the American Heart Association recommends maintaining a sodium intake of <1500 mg·day^−1^ (or <3.75 g·day^−1^ of salt) for the management of hypertension [125]. This recommendation has been shown to reduce blood pressure in both healthy and hypertensive individuals [126]. To the best of our knowledge, only one study has investigated the influence of sodium intake on blood pressure in individuals undergoing GC therapy. In this randomized, cross-over investigation, blood pressure did not change when participants shifted their salt intake to <3 or >6 g·day^−1^ (equating to 1200 or 2400 mg·day^−1^ of sodium) for a period of 3 weeks [127], indicating that sodium manipulation alone might be insufficient to influence blood pressure in these patients. This study was, however, relatively short in duration in comparison to other investigations of dietary approaches to manage hypertension, which typically last 5 weeks or more [128], and the sample investigated (*n* = 49) might have been insufficient to detect the relatively small blood pressure changes that are expected in response to sodium restriction [128]. Therefore, larger and longer studies might be required to confirm whether sodium management alone is capable of influencing GC-induced hypertension (Table 3). Pending such information, it seems prudent to recommend more holistic dietary approaches to hypertension management, in addition to adhering to sodium intake recommendations. For example, Dietary Approaches to Stop Hypertension (DASH) recommendations have proven efficacious in improving blood pressure in patients with hypertension in the general population [129]. This dietary approach consists of increasing the intake of fruits, vegetables and grains, with a balanced intake of fats, sodium and sweets [129] and, as such, aligns well with our aforementioned recommendations of basing the diet on unprocessed and minimally processed foods and reducing ultra-processed food intake. Indeed, high consumption of ultra-processed food has previously been associated with hypertension [130]; therefore, these recommendations might contribute to the alleviation of multiple adverse effects of GC therapy. It is also important to highlight that concerns have previously been raised regarding adherence to very restrictive nutritional recommendations for patients undergoing GC therapy (e.g. following very low-sodium diets) [131]. Our recommendation is that individuals maintain sodium levels within appropriate limits (∼1500 mg·day^−1^), reinforcing that this can be achieved both by reducing intake of sodium-rich, ultra-processed foods, while also reducing added salt to food preparations to a reasonable amount per day (Table 2). It is important to mention that complete elimination of added salt to the diet is unnecessary, and that it should be used primarily during the preparation of home-cooked, healthy meals.

## Behavioural considerations for implementing nutritional recommendations

The recommendations made herein are based on current evidence about how nutritional factors may prevent or reduce the occurrence of adverse effects commonly associated with GC therapy. It is important to highlight, however, that simply understanding nutritional benefits is rarely sufficient to change eating choices and habits [132, 133], and that behavioural and societal factors should also be considered. Eating is driven by a complex interplay of physiological mechanisms, genetics, epigenetics, socioeconomic and behavioural factors [134–136], in addition to disease treatment. Furthermore, food comprises more than its chemical and organoleptic characteristics; it also represents pleasure, community, family, spirituality, relationship with the world and identity expression [137–139]. Understanding why and how people eat is as important as knowing what and how much is eaten [133, 140], and we recommend that health-care professionals consider behavioural approaches to dietary adaptations. This might be particularly relevant for patients who are undergoing GC therapy, given that these medications are known to influence physiological mechanisms related to eating behaviours (e.g. hormone action, appetite, energy expenditure, reserve tissues, glucose metabolism) [73]. These factors might create a strong internal drive to eat foods that might not align with the recommendations made herein (e.g. highly palatable foods, such as ultra-processed options that are high in added fats, sugars and/or salt). Thus, nutritional interventions should consider biopsychosocial and emotional cues [133, 141] and appreciate that sensations of hunger, satiety, appetite and pleasure are influential in each individual’s food choices [132]. In this regard, the construction of an eating plan alongside the patient can be a good strategy for healthy eating, allowing patients to organize their routine and create strategies to change eating behaviours, from shopping to preparing and eating meals [142]. Moreover, collaborative goal setting, educational booklets and nutritional consultation can all be useful in supporting the patient to make real and consistent dietary changes [133, 141].

## Conclusion

In summary, GC therapy aims to harness the natural anti-inflammatory actions of these corticosteroids. Despite its clinical applications, its use can also bring about many adverse effects, including muscle and bone loss, weight gain and visceral fat accumulation, lipid and glucose dysregulation, and fluid and electrolyte imbalances. Targeted nutritional strategies, including adequate intake of high-quality protein, optimizing calcium and vitamin D status, and basing the diet on unprocessed or minimally processed food sources, while simultaneously minimizing ultra-processed food intake (Table 1), might contribute to the alleviation of these adverse effects.

It is important to highlight that although theoretically justified, many of our recommendations are based on data generated from populations who face similar challenges to patients undergoing GC therapy, and further population-specific research is required. There is currently a dearth of investigations designed specifically to test whether nutritional intervention can induce real-life clinical improvements in individuals undergoing GC therapy, and important research questions remain unanswered (Table 3). As such, the efficacy of these nutritional recommendations, in addition to the viability of their implementation, should be assessed using cross-sectional observational studies, large cohort studies and high-quality randomized controlled clinical trials. It is also important to consider that patients undergoing GC therapy are a heterogeneous population and might present with specific nutritional needs other than the ones presented. The recommendations provided herein should, therefore, be considered as general guidelines, which might warrant adaption in accordance with individual requirements, preferences and goals.

## Supplementary Material

rkac029_Supplementary_Data

## Data Availability

No new data were generated or analysed in support of this research.
