# Supplementary material for: Nutritional recommendations for patients undergoing prolonged glucocorticoid therapy
Source: Rheumatol Adv Pract. 2022 Apr 21;6(2):rkac029. doi: 10.1093/rap/rkac029 (PMC9080102; doi:10.1093/rap/rkac029)

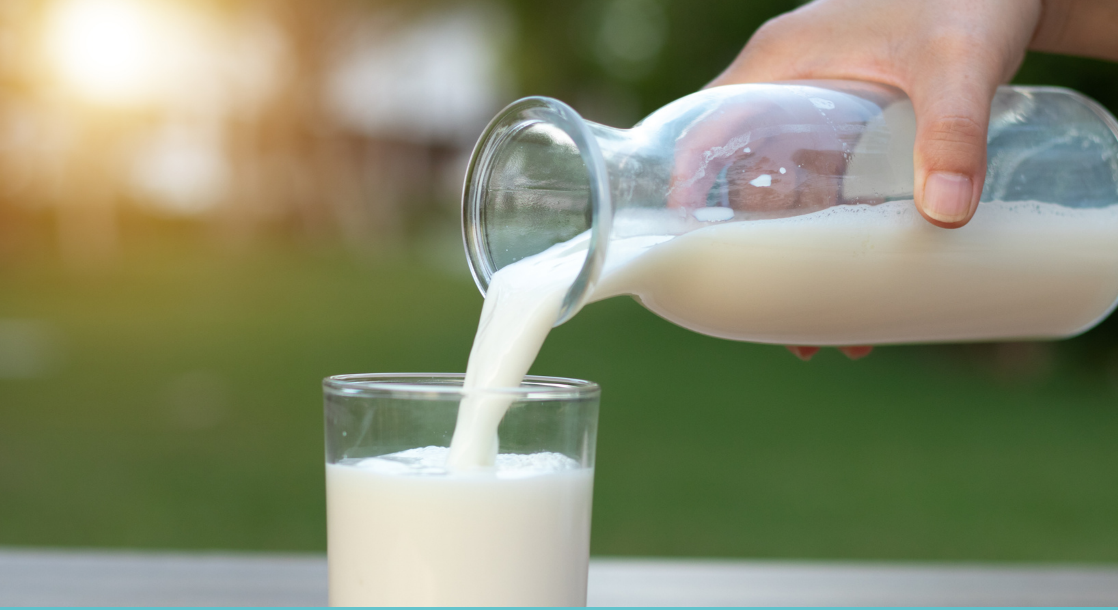

NUTRITIONAL RECOMMENDATIONS FOR PATIENTS UNDERGOING GLUCOCORTICOID THERAPY

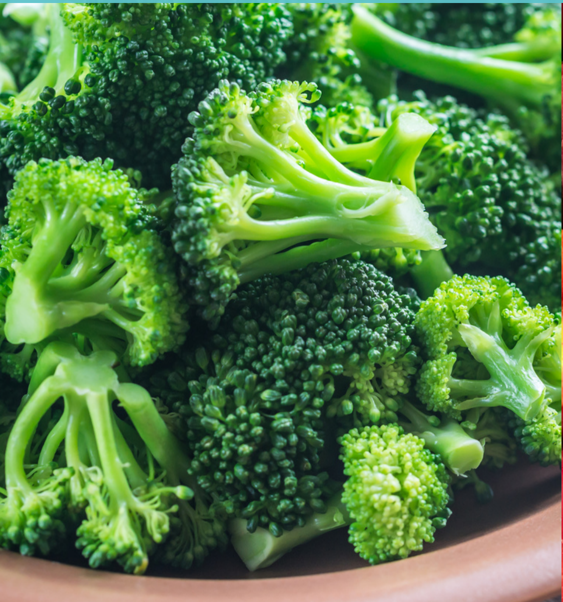

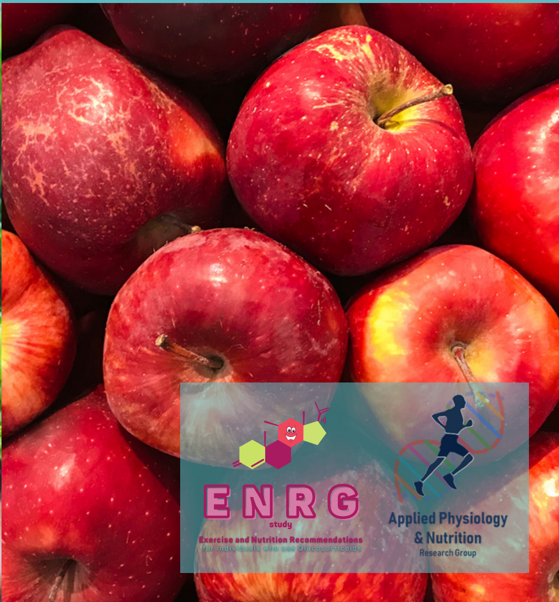

ENRG study

Exercise and Nutrition Recommendations for individuals who use Glucocorticoids

Applied Physiology & Nutrition Research Group

Getting to know *food processing* categories

A healthy diet should be balanced, rich in natural and unprocessed foods, and contain few ultraprocessed products. But, do you know how to tell them apart?

Currently, many food guides recommend that we eat **MORE** unprocessed foods, and **AVOID** ultraprocessed foods. This dietary pattern is related to a healthier lifestyle, and can possibly lower risk of many diseases, and possibly improve your health when taking glucocorticoids.

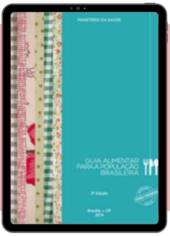

Unprocessed or minimally processed foods

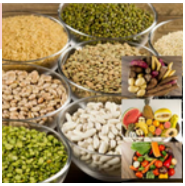

These foods should be the basis of our diet. They go through either no processing or very minimal processing, such as cleaning and pasteurizing. Rice, beans, pasta, eggs, milk, fruits, vegetables, and meats are the main components of this category.

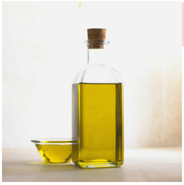

Culinary ingredients (Oils, fat, salt and sugar)

Ingredients from this group should be used carefully and in moderation to season and cook other foods, creating nutritious and delicious recipes.

Processed foods

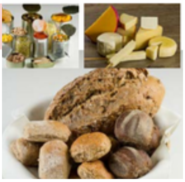

Foods in these group are made by combining natural foods with culinary ingredients, such as bread, made with flour, salt, and oil; cheeses, made from milk and salt; and preserved vegetables, made from natural vegetables and preserving agents. Consumption of these foods should be moderate.

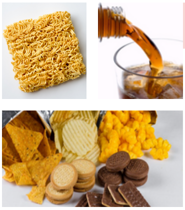

Ultraprocessed foods

These foods should be avoided and be minimally present in our diet. They're made by the industry through large amounts of added salt, sugar, fats, chemical flavoring and dyeing agents. Even though these foods are convenient, they're commonly nutritionally unbalanced and can be harmful to our health. Examples of this group are sodas, powdered fruit juices, snacks and candies, and other instant or packaged meals.

How can diet help with the *adverse effects* of glucocorticoid therapy?

Glucocorticoid therapy can lead to adverse effects, such as:

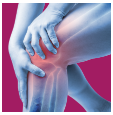
Increased risk of bone fracture

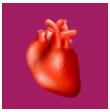
Increased risk for cardiovascular diseases

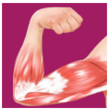
Muscle weakning

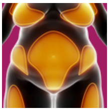
Weight gain

Improving your **dietary habits** can be useful to reduce these adverse effects and improve quality of life.

Calcium

Calcium (Ca) is a mineral responsible for many functions in the body, but mainly for building strong bones.

Recommended daily intake

Aim for 1,000–1,200 mg per day

4 to 5 portions of calcium-rich foods

Calcium-rich foods		Meal
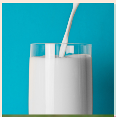	**Whole cow milk**<br>1 cup (240 ml)<br>*Ca: 280 mg*	**Breakfast and snacks**
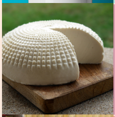	**Mozzarella cheese**<br>1 and 1/2 slices (50 g)<br>*Ca: 350 mg*	**Breakfast and snacks**
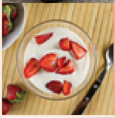	**Whole yogurt**<br>1 cup (240 g)<br>*Ca: 290 mg*	**Breakfast and snacks**
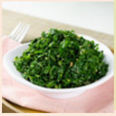	**Cooked dark leafy greens**<br>2 tablespoons (42 g)<br>*Ca: 60 mg*	**Lunch and dinner**

Source: U.S. Department of Agriculture FoodData Central

Vitamin D

An essential nutrient that functions as a hormone, improving and maintaining bone health.

Recommended daily intake

600–1000 UI per day

2 to 3 portions of foods rich in vitamin D

Vitamin D can be either produced by the body, following sunlight exposure, or acquired by the diet.
If you have a skin condition, or have poor sunlight exposure in your daily routine, make sure to ingest it via your diet!

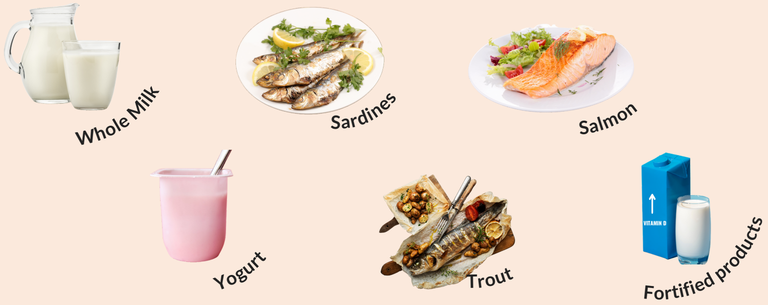

Dietary sources of vitamin D

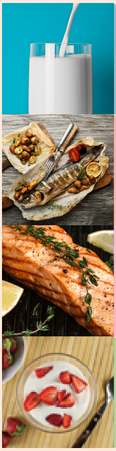

Whole milk (fortified)
1 cup (240 g)
Vit. D: 90 UI

Trout
3 ounces (85 g)
Vit. D: 650 UI

Salmon
3 ounces (85 g)
Vit. D: 383 UI

Skimmed Yogurt
8 ounces (220 g)
Vit. D: 116 UI

Source: U.S. Department of Agriculture FoodData Central

Protein

Protein is essential for creating and maintaining healthy and strong muscles.

A few options are:

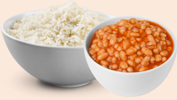

Rice and Beans

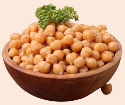

Chickpeas

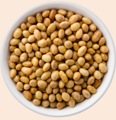

Soybeans

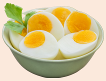

Eggs

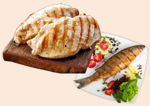

Chicken or fish

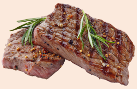

Meat

Recommendation

Varies according to individual needs, but aim for at least two portions of protein-rich foods per day.

Sodium

Sodium is an essential mineral that regulates blood pressure and fluids throughout the body. During glucocorticoid use, blood pressure might increase, and sodium intake must be moderated.

However, **it should not be excluded entirely from the diet**, but rather consumed **in a balanced amount.**

PREFER

Preparing your own meals, without adding too much salt, and using natural and flavorful ingredientes

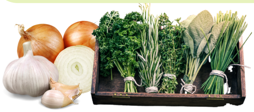

- ONION
- GARLIC
- FRESH AND DRIED HERBS
(basil, rosemary, thyme, parsley, cilantro, green onion)

AVOID

- Adding too much salt in meals

- Consuming ultraprocessed foods
(exemples: instant pasta, salty snacks)

- Consuming other products rich in sodium
(examples: industrialized sauces and vegetable/meat stock cubes)

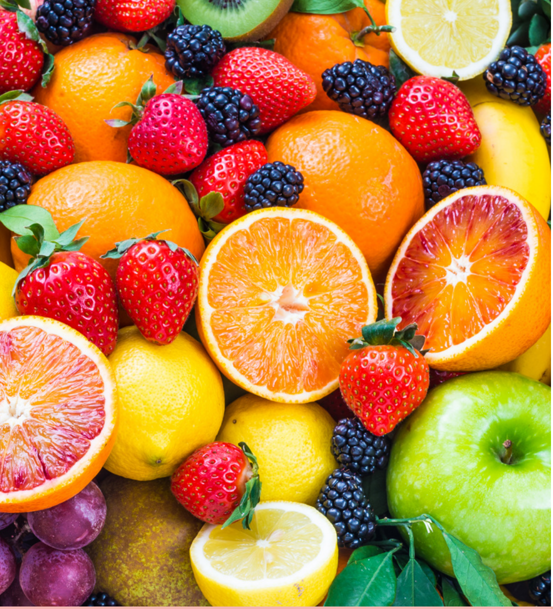
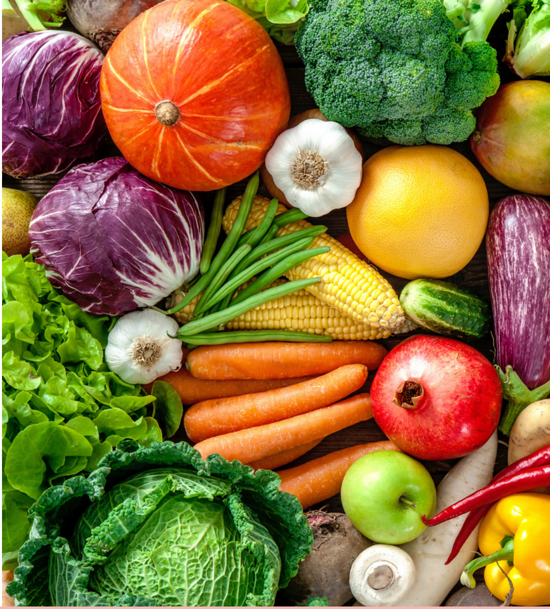

Different people have different needs!

Regularly check with your doctor or a registered dietitian nutritionist to figure out the best recommendations for you!

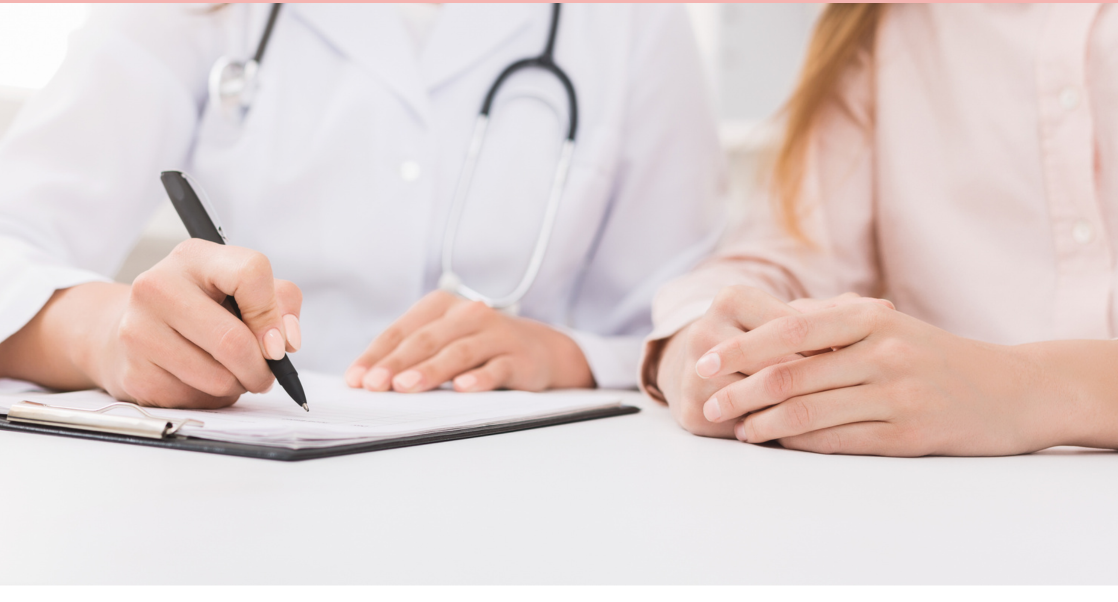

Supplement: rkac029_Supplementary_Data [file rkac029_supplementary_data.pdf]
